# Determinants of stillbirths in Northern Ghana: a case control study

**DOI:** 10.11604/pamj.supp.2016.25.1.6168

**Published:** 2016-10-01

**Authors:** Adam Bukari Badimsuguru, Kofi Mensah Nyarko, Edwin Andrew Afari, Samuel Oko Sackey, Chrysantus Kubio

**Affiliations:** 1 Ghana Field Epidemiology and Laboratory Training Programme, Department of Epidemiology and Disease Control, School of Public Health, College of Health Sciences, University of Ghana, Legon, Ghana; 2 Ghana Health Service, Northern Regional Health Directorate, Tamale, Ghana; 3 Ghana Health Service, West Gonja District Health Directorate, Damongo, Ghana

**Keywords:** Stillbirth, determinants, case control, study, Tamale Metropolitan Area

## Abstract

**Introduction:**

Stillbirths are more common than the death of a baby after birth. In 2012, Tamale Metropolitan Area in the Northern Region of Ghana reported 35 stillbirths per 1,000 deliveries. This study was therefore conducted to determine the sociodemographic, obstetric and maternal medical health related risk factors associated with stillbirths.

**Methods:**

A 1:1 unmatched case control study was conducted in the Tamale Metropolis. Cases were defined as singleton lifeless babies delivered by resident mothers in Tamale Metropolis at or after 28 weeks of gestation from 1st January, 2012 to 31st December, 2013. Controls were those who had live babies within the same period. We abstracted data from maternal health record booklets used in index pregnancies. We also conducted personal interviews with mothers on home visits. We estimated both crude and adjusted odds ratios, 95% confidence intervals and p values.

**Results:**

A total of 368 mothers (184 cases and 184 controls) participated in the study. Maternal age of ≤ 24 years, prolonged labour (> 12 hours) and diastolic blood pressure of less than 80mmHg in late pregnancy were significant determinants of stillbirths (aOR = 3.0, 95% CI 1.08 – 8.39; aOR = 3.5, 95% CI 1.94 – 6.61; aOR =2.2, 1.04 – 4.54 respectively).

**Conclusion:**

Low diastolic blood pressure in late pregnancy, young maternal age and prolonged labour were the key determinants of stillbirths in the Tamale Meetropolis. Improvement of community moral practices and discouraging early marriage will help reduce the menace of stillbirths. Monitoring of blood pressure and labour should be prioritized.

## Introduction

Stillbirth is a baby born with no sign of life at or after 28 completed weeks of gestation. The death of a foetus in utero or at birth is a devastating experience to the affected mothers and families. Stillbirth is an indicator of access to and quality of obstetric care, including utilization of services in a geographic location. Globally, stillbirths are steadily increasing and accounts for more than 50% of perinatal mortality [1, 2]. The rates of stillbirth in developing countries are four to ten times compared to the developed nations, however, the incidence rates are highest in sub Saharan Africa [3]. In Ghana, although the rate of stillbirth is high ranging between 13 and 22 per 1,000 births [4, 5], the highest rate is in the northern part of the country. In 2012, the Tamale Metropolitan Area which is in the northern region of Ghana reported an annual stillbirth rate of 35 per 1,000 births [6]. The global “big five” causes of stillbirths are childbirth complications, maternal infections in pregnancy notably syphilis, maternal conditions especially hypertension and diabetes, fetal growth restriction and congenital abnormalities [7]. The risk factors and other causes associated with stillbirth differ substantially in developing countries [8]. A cross sectional study conducted in the Ashanti Region of Ghana found malaria and anaemia to be risk factors of stillbirth [9]. Also in the Upper East Region of Ghana, the risk factors associated with perinatal mortality were birth injuries, prematurity and infections [10]. The previous studies on stillbirths have all been descriptive in nature and therefore the need to conduct analytical studies on the determinants of the key risk factors. The objectives of this study were to assess the obstetrics, maternal medical conditions and sociodemographic determinants of stillbirths amongwomen in the Tamale Metropolitan Area.

## Methods

An unmatched case control study was conducted in the Tamale Metropolitan Area in the Northern region of Ghana. The case control ratio was 1:1. The Tamale Metropolis is located in the heart of Northern region of Ghana with an estimated population of 371,351 (185,995 males and 185,356 females). It is one of the fastest growing metropolitan area in the West Africa sub-region. The study population included resident women of Tamale Metropolitan area who delivered in Tamale Teaching Hospital and/or Tamale West Hospital. Tamale Teaching Hospital is a tertiary referral centre while Tamale West Hospital is a secondary level service point with labour and maternity wards. These health facilities provide Comprehensive Emergency Obstetric and Neonatal Care (EmONC). Other government health facilities and private health facilities exist and provide basic reproductive health services. Only mothers who had singleton births were included in the study. The required sample size of 368 women comprising 184 mothers of stillborn babies (cases) and 184 mothers of live born babies (controls) was calculated using Fleiss formula. Mothers (cases and controls) were initially identified in the Labour Wards delivery registers in the Tamale Teaching Hospital and the Tamale West Hospital. Cases included mothers who delivered singleton stillborn between 1st January, 2012 and 31st December, 2013 and resident in the Tamale Metropolitan area. Controls were mothers who had singleton live births between 1st January, 2012 and 31st December, 2013 and resident of Tamale Metropolitan area. Cases were randomly selected from the list of cases (sample frame) of the hospitals. The number of cases selected in each facility was proportional to their caseload. We replaced those who could not be located or refused to participate from the lists created. A similar approach was used in the control selection from these facilities. Structured questionnaire was administered to collect socio demographic characteristics, which included age, height, residence, education and occupation. Obstetric variables collected were parity, birth interval, gestation, and mode of delivery, presentation of the foetus, duration of labour, sex and birth weight. Maternal medical characteristics included diagnosis of malaria in the index pregnancy, blood pressure, haemoglobin levels, protein and sugar in urine. Also, we visited mothers in their homes and abstracted antenatal records from their maternal health record books used during the index pregnancy. The research assistants conducted face to face interview with the mothers during home visits to complete parameters that were not documented. Research assistants and data entry clerks were trained on the study protocol and supervisors also randomly checked completed data forms to identify errors and omissions and corrective actions taken. The data was coded and double entered into a predesigned template in Epi Data. The data were then exported to STATA version 11.0 software for analysis. Univariate analysis of demographic characteristics was performed using frequencies and percentages. Odds ratios, 95% confidence intervals and p values were estimated to identify associations between independent variables and stillbirth. Significance level was set at a p-value less than 0.05. The variables which were significant as well as those proven to be risk factors of stillbirths were put in a multivariate logistic regression model to detect independent determinants. The Ethical Review Committee of Ghana Health Service reviewed and approved the study protocol before its implementation. Also, permissions to conduct the study in the Tamale Metropolitan Area was obtained from the following institutions: Tamale Metropolitan Health Administration, Tamale Teaching Hospital and Tamale West Hospital. Informed consent was obtained from each participating mother who met the inclusion criteria and agreed to participate in the study. The questionnaire was piloted in an adjoining district, the Savelugu/Nanton district.

## Results

### Demographic characteristics of cases and controls

A total of 368 mothers consisting of 184 stillborn mothers and 184 live born mothers participated in the study. Table 1 shows the demographic characteristics of the study participants. Out of the 368 mothers, those aged 25 – 34 years were, 189 (51.4%), while mothers between 35 and 46 years were lowest frequency 77 (20.9%). The proportion of stillborn mothers aged 24 years or less was 25.0% while mothers aged 35 years or more formed 21.2%. Controls aged 24 years or less were 30.4% while 20.7% were 35 years or more. Fifty-nine (17.3%) of the mothers were less than 150cm in height. About 21% of the cases were under 150cm compared to 13.2% of the controls. Mothers living in the rural area were 185 (50.3%) compared to mothers in the urban communities, 183 (49.7%). Comparing cases to control by residence, 100 (54.3%) cases resided in rural areas compared to 85 (46.2%) controls.

**Table 1:** Demographic characteristic of cases and controls

Characteristic	Cases (%)	Controls (%)	Total (%)
	N = 184	N = 184	368
**Age (years)**			
≤ 24	46 (25.0%)	56 (30.4%)	102(27.7%)
25 – 34	99 (53.8%)	90 (48.9%)	189 (51.4%)
35 – 46	39 (21.2%)	38 (20.7%)	77 (20.9%)
**Height (cm) (341)**			
< 150	38 (20.9%)	21 (13.2%)	59 (17.3%)
≥ 150	144(79.1%)	138 (86.8%)	282 (82.7%)
**Residence**			
Urban	84 (45.7%)	99 (53.8%)	183 (49.7%)
Rural	100 (54.3%)	85 (46.2%)	185 (50.3%)

### Sociodemographic determinants of stillbirths

Maternal age was found to be the only demographic determinant significantly associated with stillbirth in the multivariate model, though in the bivariate analysis it was not significant (Table 2). Mothers aged = 24 years were at high risk of stillbirth compared to mothers of age between 25 – 34 years (aOR = 3.0, 95% CI 1.08 – 8.39). Maternal height and residence were not associated with stillbirth as shown in Table 2. We did not find any significant socioeconomic determinant in this study. Mothers with primary education or no education had lower risk of stillbirth compared to mothers with tertiary education (cOR = 0.2, 95% CI 0.04 – 0.53; cOR = 0.3 95% CI 0.09 – 0.96 respectively). These protective effects disappeared in the multivariate model (Table 2). Mothers who were engaged in trade and those employed had high risk of stillbirth compared to housewives (cOR = 2.2, 95% CI 1.32 – 3.49; cOR = 3.4, 95% CI 1.38 – 8.55 respectively). The associations disappeared at the multivariate level (aOR = 1.4, 95% CI 0.67 – 3.01; aOR = 2.5, 0.27 – 23.85 respectively). Farmers wives were less likely to have stillbirths compared to mothers whose husbands were on other employments (cOR = 0.5, 95% CI 0.25 – 0.87), but this association was not significant in the multivariate model (aOR = 0.6, 95% CI (0.23, 1.89). The other variables were not associated with stillbirth (Table 2).

**Table 2:** Sociodemographic determinants of stillbirths among cases and controls

	Cases (%)	Controls (%)	cOR (95% CI)	p value	aOR (95% CI)	p value
**Determinant**	**N = 184**	**N = 184**				
**Maternal age (years)**				0.490		**0.026**
25 - 34	99 (53.8%)	90 (48.9%)	1		1	
≤ 24	46 (25.0%)	56 (30.4%)	1.3 (0.82,2.17)		3.0(1.08,8.39)	
35 - 46	39 (21.2%)	38 (20.7%)	1.1 (0.63,1.82)		1.1(0.55,2.23)	
**Height (cm)**				0.062		
≥ 150	144 (79.1%)	138 (86.8%)	1			
< 150	38 (20.9%)	21 (13.2%)	0.5 (0.32, 1.03)			
**Residence**				0.117		
Urban	84 (45.7%)	99 (53.8%)	1			
Rural	100 (54.3%)	85 (46.2%)	0.7 (0.47, 1.08)			
**Mother education level**				**0.004**		0.297
Tertiary	4 (2.2%)	13 (7.1%)	1		1	
Secondary	28 (15.2%)	38 (20.7%)	0.4 (0.12, 1.42)		1.2 (0.11,12.25)	
Primary	35 (19.0%)	17 (9.2%)	0.2 (0.04, 0.53)		0.6(0.05,8.50)	
No education	117 (63.6%)	116 (63.0%)	0.3 (0.09, 0.96)		1.1(0.08,12.35)	
**Mother occupation**				**0.002**		0.270
House wife	62 (33.7%)	34 (18.5%)	1		1	
Trade	113 (61.4%)	133 (72.3%)	2.2 (1.32,3.49)		1.4(0.67,3.01)	
Employed	9 (4.9%)	17 (9.2%)	3.4 (1.38,8.55)		2.5(0.27,23.85)	
**Husband occupation**				**0.011**		**0.091**
Employed	29 (15.8%)	36 (19.6%)	1		1	
Trade	85 (46.2%)	106(57.6%)	1.0 (0.57,1.76)		1.2(0.48,3.13)	
Farmer	69 (37.5%)	40 (21.7%)	0.5 (0.25,0.87)		0.6(0.23,1.89)	
No work	1 (0.5%)	2 (1.1%)	1.6(0.14,18.66)		0.7(0.02,24.85)	

### Obstetric determinants associated with stillbirths

Prolonged labour and birth weight were found to be significant (Table 3). Mothers who labored for >12 hours had high risk of stillbirth compared to those who delivered within 12 hours of labour (aOR = 3.5, 95% CI 1.94, 6.61). This was also observed in the bivariate model (cOR = 4.7, 95% CI 3.04 – 7.33). In the bivariate analysis, birth weight of < 2.5kg had lower risk of stillbirth compared to birth weight range between 2.5kg and 3.9kg (cOR = 0.2, 95% CI 0.09 – 0.32). The effect was marginally reduced in multivariate analysis but still remained significant (aOR = 0.3, 95% CI 0.12 – 0.74). Birth interval of < 24 months had increased risk of stillbirth compared to birth interval of = 24 months (cOR = 2.6, 95% CI 1.29 – 5.69) as shown in Table 3. In the multivariate analysis, birth interval was not a significant determinant of stillbirth (aOR = 2.0, 95% CI 0.79 – 5.19). Parity, gestation and mode of delivery were not significantly associated with stillbirth (Table 3).

**Table 3:** Obstetric determinants and risk of stillbirth among 184 cases and 184 controls in the Tamale Metropolitan area, 2012 - 2013

	Cases (%)	Controls (%)	cOR (95% CI)	p value	aOR (95% CI)	p value
**Deteiminant**	N = 184	N = 184				
Parity				**0.443**		
0	49 (26.6%)	41 (22.3%)	1			
1	34 (18.5%)	43 (23.4%)	1.5(0.82,2.78)			
2 -3	58 (31.5%)	64 (34.8%)	1.3(0.76,2.77)			
> 4	43 (23.4%)	36 (19.6%)	1.0(0.54,1.83)			
Birth interval in months (n = 283)				**0.003**		**0.140**
> 24	108 (77.7%)	130 (90.3%)	1		1	
< 24	31 (22.3%)	14 (9.7%)	2.6(1.29,5.69)		2.0 (0.79, 5.19)	
**Pregnancy gestation at birth (weeks)**				**0.341**		
> 37	103 (56.0%)	112 (60.9%)	1			
< 37	81 (44.0%)	72 (39.1%)	0.8(0.54,1.24)			
**Mode of delivery**				**0.069**		**0.059**
SVD	134 (72.8%)	152 (82.6%)	1		1	
C/S	49 (26.1%)	30 (16.3%)	0.5(0.33,0.92)		0.5 (0.23, 1.18)	
Vacuum	2 (1.1%)	2 (1.1%)	0.8(0.12,6.34)		0.3 (0.03, 2.64)	
**Labour duration (hours)**				**0.000**		**0.000**
< 12	60 (32.6%)	128 (69.6%)	1		1	
>12	124 (67.4%)	56 (30.4%)	4.7(3.04,7.33)		3.5 (1.94, 6.61)	
**Birth weight (kg)**				**0.000**		**0.006**
2.5 -3.9	108 (58.7%)	156 (84.8%)	1		1	
< 2.5	66 (35.9%)	17 (9.2%)	0.2(0.09,0.32)		0.3 (0.12, 0.74)	
>4.0	10 (5.4%)	11 (6.0%)	0.7(0.31,1.85)		1.0 (0.29, 3.16)	

### Maternal medical health related factors

Low diastolic blood pressure of less than 80mmHg, especially in late pregnancy was significantly associated with stillbirth (aOR = 2.2, 95% CI 1.04 – 4.54). A similar observation was made in the bivariate model (cOR = 31, 95% CI 1.84 – 5.16). In the bivariate analysis, systolic blood pressure of = 99mmHg and diastolic blood pressure of < 80mmHg at registration had increased risk of stillbirth compared to systolic blood pressure range of 100 – 139mmHg and diastolic blood pressure range of 80 – 85mmHg (cOR = 1.8, 95% CI 1.02 – 3.29 and cOR = 3.2, 95% CI 1.77 – 5.66 respectively). These associations disappeared in the multivariate model (Table 4).

**Table 4:** Maternal medical related determinants of stillbirth among 368 mothers in the Tamale Metropolitan area, 2012 - 2013

	Cases (%)	Controls (%)	cOR(95% CI)	p value	aOR (95% CI)	p value
**Determinant**	N = 184	N = 184				
**Malaria in pregnancy**				**0.166**		
No	149 (81.0%)	138 (75%)	1			
Yes	35 (19.0%)	46 (25%)	1.4 (0.86, 2.33)			
**Haemoglobin level at 36 weeks (g/dl) (N = 287)**				**0.576**		
≥ 9.5	104 (66.2%)	82 (63.1%)	1			
< 9.5	53 (33.8%)	48 (36.9%)	1.2 (0.71, 1.86)			
**Systolic BP (mmHg) at registration (N = 367)**				**0.039**		**0.59**
100 - 139	163 (88.6%)	148 (80.9%)	1		1	
≤ 99	21 (11.4%)	35 (19.1%)	1.8 (1.02, 3.29)		1.3 (0.54, 2.98)	
**Diastolic BP (mmHg) at registration (N = 367)**				**0.000**		**0.055**
80 - 85	48 (26.1%)	19 (10.4%)	1		1	
86 - 90	7 (3.8%)	2 (1.1%)	0.7 (0.14, 3.79)		0.2 (0.00, 5.58)	
< 80	129 (70.1%)	162 (88.5%)	3.2 (1.77, 5.66)		1.9 (0.79, 4.83)	
**Systolic BP (mmHg) at 36 weeks (N = 361)**				**0.012**		–
100 -139	157 (87.7%)	158 (86.8%)	1		1	
140 -159	10 (5.6%)	2 (1.1%)	0.2 (0.04, 0.92)		0.1 (0.00, 1.23)	
≤ 99	12 (6.7%)	22 (12.1%)	1.8 (0.87, 3.81)		2.3 (0.77, 7.12)	
**Diastolic BP (mmHg) at 36 weeks (N = 361)**				**0.000**		**0.004**
80 - 85	58 (32.4%)	30 (16.5%)	1		1	
86 - 90	27 (15.1%)	8 (4.4%)	0.6 (0.23, 1.41)		1.0 (0.24, 4.32)	
91 -120	5 (2.8%)	2 (1.1%)	0.7 (0.14, 4.22)		31.5 (0.52, 190)	
< 80	89 (49.7%)	142 (78.0%)	3.1 (1.84, 5.16)		2.2 (1.04, 4.54)	

Mothers with slightly raised systolic blood pressure (140 – 159mmHg) showed a lower risk of stillbirth compared to mothers with blood pressure between 100 – 139mmHg at 36 weeks (cOR = 0.2, 95% CI 0.04 – 0.92). The effect disappeared in the multivariate model (aOR = 0.1, 95% CI 0.00 – 1.23). Malaria in pregnancy and anaemia in late pregnancy were not significant determinants (cOR = 1.4, 95% CI 0.86 – 2.33; cOR =1.2, 95% CI 0.71 – 1.86 respectively).

## Discussion

In this unmatched case control study, we assessed sociodemographic, obstetric and maternal medical related determinants of stillbirths in the Tamale Metropolitan Area. The result of the study revealed that maternal age of =24 years, prolonged labour (>12 hours) and diastolic blood pressure of <80mmHg at 36 weeks as significant determinants of stillbirths in the Tamale Metropolitan Area. Low birth weight (<2.5kg) reduced the risk of stillbirth. The extremes of reproductive age spectrum substantially conferred risk of stillbirth to mothers who fell within these categories of ages. Our results suggest that mothers who were 24 years or less had a three folds elevated odds of stillbirth compared to mothers in the age brackets of twenty five to thirty four years. Other studies have demonstrated that early child bearing is significantly associated with stillbirth [11]. However, our study result was not consistent with what other studies found in the United States of America [12]. This could be that, young women in the developed countries may be well built due to good nutrition and also having access to good health care. Prolonged labour (= 12 hours) was the most significant obstetric determinant of stillbirth. The study finding was consistent with other studies who reported prolonged labour among singleton births in a slum population in Bangladesh [13] and Pakistan [14]. Many mothers still attempt home delivery or delayed in decision to seek skill delivery and this can result in late intervention of prolonged labour. Our finding shows that low birth weight had lower risk for stillbirth compared to normal birth weight (2.5 – 3.9kg). Recent studies have shown that low birth weight is associated with neonatal death than stillbirth due to immaturity, hypothermia, hunger and low immunity. Stillbirths in normal birth weight may be related to foetal infection in late pregnancy [15] as a result of deficient prenatal care. Although hypertension is a known risk factor of stillbirth, our results showed that women whose blood pressure falls in the borderline hypotensive range appear to be at high risk of stillbirth than those women in the normal group. This is similar to a study conducted by Warland and colleagues in Australia [16]. Our study had some limitations which should be taken into account in interpreting these results. This was a retrospective study and we were uncertain about the caliber of personnel who recorded the data as well as the accuracy of tools used to estimate the parameters. We also anticipated some degree of recall bias, especially those who delivered in early 2012.

## Conclusion

Mothers who are 24 years or less may have greater odds of stillbirth in the Tamale Metropolitan area. Prolonged labour is more likely to be a contributing determinant of stillbirth. Low birth weight was found to be protective. Borderline diastolic hypotension in late pregnancy may significantly increase the mothers’ odds of stillbirth. The knowledge this study adds is that lower borderline hypotension is a strong determinant of stillbirth. The Metropolitan Health Administration should involve religious and opinion leaders to educate the community on the need for early reporting to health facilities to avoid prolonged labour. Pregnancies with normal foetal weight should be given equal attention like those with extreme weight as the study has found that normal weight foetuses are also at risk. Midwives and Community Health Officers should monitor the blood pressure of pregnant mothers in late pregnancy.
